# Applying ACE-III, M-ACE and MMSE to Diagnostic Screening Assessment of Cognitive Functions within the Polish Population

**DOI:** 10.3390/ijerph191912257

**Published:** 2022-09-27

**Authors:** Beata Kaczmarek, Zofia Ilkowska, Sylwia Kropinska, Sławomir Tobis, Roma Krzyminska-Siemaszko, Aleksandra Kaluzniak-Szymanowska, Katarzyna Wieczorowska-Tobis

**Affiliations:** 1 Department of Palliative Medicine, Poznan University of Medical Sciences, 61-245 Poznan, Poland; 2 Department of Occupational Therapy, Poznan University of Medical Sciences, 60-781 Poznan, Poland

**Keywords:** cognitive screening tests, Addenbrooke’s cognitive examination, mini-Addenbrooke’s cognitive examination, mini-mental state examination

## Abstract

The research aims to compare the accuracy of the mini-mental state examination (MMSE), the Addenbrooke’s cognitive examination III (ACE-III) and the mini-Addenbrooke’s cognitive examination (M-ACE) within the Polish population. The model comprised several stages: the features of each test were compared; the shifts in result categorisations between the norm and below the norm were analysed; a third category—mild cognitive impairment (MCI)—was included. Additionally, particular ACE-III domains that scored below domain-specific norm thresholds were analysed to establish the potential early predictors of dementia. All tests correlated to a high and very high degree—cf. MMSE and ACE-III (*r* = 0.817; *p* < 0.001), MMSE and M-ACE (*r* = 0.753; *p* < 0.001), ACE-III and M-ACE (*r* = 0.942; *p* < 0.001). The area under the ROC curve for the ACE-III diagnostic variable had a high value (AUC = 0.920 ± 0.014). A cut-off point of 81 points was suggested for ACE-III; the M-ACE diagnostic variable had an equally high value (AUC = 0.891 ± 0.017). A cut-off point of 20 points was suggested. A significant decrease in the mean score values for people who scored norm or below the norm under ACE-III, as compared to the MMSE results for norm (*p* < 0.0001), occurred for speech fluency (which decreased by 26.4%). The tests in question are characterised by high sensitivity and specificity. Targeted ACE-III seems best recommended for use in specialised diagnostic centres, whereas M-ACE appears to be a better suited diagnostic alternative for primary health care centres in comparison to MMSE.

## 1. Introduction

Early diagnosis of cognitive disorders in older people is a prerequisite for any therapy and activation programmes. The American Academy of Neurology recently recommended annual tests for cognitive disorders in patients above 65 who are under neurological care [1]. Additionally, the Alzheimer’s Association suggests that screening tests for cognitive disorders be repeated at least once a year, including for patients with memory deficits, as well as those who declare functioning problems, which could stem from other cognitive disorders, and emotional functioning problems. Moreover, the study lists the recommended screening tools. Ideally, a screening test should last about five minutes and be valid for primary and specialist health care units. It should also be easily administrable by medical personnel (including non-physicians), and additionally, it should display accuracy in psychometrics (high sensitivity and specificity, low error rate) and include unbiased content in terms of language, education and culture. Lastly, it should be free. Building a universal diagnostic method, which fulfils those conditions, presents an enormous challenge.

The World Health Organization are currently working on their own recommendations; however, all the documents published so far stress the validity of annual cognitive tests in people 65+ as part of primary health care services. Still, there has been no binding statement as to actual recommendations [2]. Poland has adopted the recommendations of the Polish Alzheimer’s Association [3], which state that screening tests should be administered by physicians, irrespective of their specialisation, psychologists, nurses and social workers who underwent dedicated training on administering and interpreting a given diagnostic toolkit. Experts concur such tests ought to be administered in patients above 65 or younger if their family’s medical history includes cases of dementia, in patients with disorders that affect cognitive functions (such as diabetes, thyroid, heart, lung or liver conditions), as well as patients who report worsened cognitive performance [3] (p.18). Mini-mental state examination (MMSE) is a testing tool recommended for its reliability, feasibility, availability and ability to state the rate of impairment. Clinical practice does not recommend using MMSE as the only diagnostic tool in screening differential diagnostics. Critics point out that the test does not exhibit enough sensitivity to differentiate a diagnosis of neurodegenerative disease other than Alzheimer’s disease (AD), including early stages of dementia [3].

A noteworthy 2019 Cochrane review defines the current meta-analysis, which pertains to methods currently used in detecting dementia and MCI, namely, Addenbrooke’s cognitive examination-III (ACE-III) and mini-Addenbrooke’s cognitive examination (M-ACE/Mini-ACE) [4]. The following overview is based on publicly available research [5,6,7,8] and clinical practice.

Applying ACE-III as a screening method and a useful tool in the differential diagnosis of dementia variants has been researched frequently. Sitek et al. [9] presented a qualitative profile analysis employing ACE-III, which is useful to any clinician in the differential diagnosis of dementia. Thus, late-onset Alzheimer’s disease (loAD) exhibits lower results for the sub-section of memory (a significantly lower performance level as compared to other tasks is characteristic of amnesic types of AD), as well as temporal and spatial orientation (initial sub-sections of attention span) or speech fluency and, to a lesser degree, language functions and visuospatial functions.

In people with episodic memory disorders, especially in postponed reproduction, and semantic memory disorders (speech fluency and naming test), there may occur amnesic MCIs with a tendency towards AD convergence [10].

M-ACE, on the other hand, is a short version of ACE-III. To verify its validity, there were 242 participants tested, including 28 with a recognised form of Alzheimer’s disease, 23 with a behavioural variant of frontotemporal dementia, 82 people with primary progressive aphasia, 21 with cortico-basal dementia and 78 people in a control group. According to Sitek et al. [9], the test in question is characterised by high sensitivity (0.85) and specificity (0.87) for cut-off points of 25 or lower and a very high likelihood of dementia for results of 21 points or lower. The test shows greater sensitivity as compared to MMSE, as well as mitigating the risk of the ceiling effect [8]. In a Spanish cohort test, where participants were low educated and of mixed dementias, including a control group, the optimal sensitivity and specificity (0.867) and specificity (0.870) were claimed for results between 16/30 and 17/30 [11]. This study constitutes a high value because of the possibility of verifying the results for their sensitivity, which can minimise the false negative diagnoses, which, in turn, could delay proper treatment, patient support and care. Additionally, the test helps to accurately diagnose both dementia and mild cognitive impairments. The M-ACE results above the cut-off points exclude both dementia and MCIs. M-ACE appears more differential towards identifying MCI than MoCA than indicated by previous comparative studies [12,13]. The high accuracy of M-ACE in diagnosing mild cognitive impairments was additionally confirmed by a meta-analysis using a statistical variable for the ‘likelihood to be diagnosed or misdiagnosed, LDM’, especially when compared with such screening tests as MMSE, MoCA or other tests used in Poland exclusively in academic facilities—six-item cognitive impairment test (6CIT) and ascertain dementia 8 (AD8) [14,15]. Moreover, research indicates that M-ACE, rather than MMSE or MoCA, appears more useful in detecting mild cognitive impairments as well as dementia when a different language version is used, which could suggest it is relatively culture neutral [16,17]. M-ACE is a quick, easy-to-use and patient-friendly tool, which provides an opportunity to diagnose MCI early on or effectively rule out dementia [18,19]. Relating to the above observations, we conducted a study whose purpose was to compare the most commonly used screening tests in Polish adaptation in terms of their sensitivity and specificity, usefulness in predicting the development of a neurodegenerative disease and identifying the cognitive deficits profiles of respondents in the Polish population.

## 2. Methods

### 2.1. Patients

The study was conducted from 2015 to 2018. Participants were referred for a psychological test by a geriatrician or other specialist residing at the geriatric health centre *WSWOP Hospicjum Domowe* in Poznań, Poland, or volunteered to take part in activation programmes for older adults and underwent cognitive screening as part of the project’s diagnostic procedure.

In total, 386 people qualified and their medical records were analysed for the study.

### 2.2. Measurements

The study was conducted using three popular cognitive ability screening tests, which were translated into Polish: MMSE—a most often applied 30-point test with international and interdisciplinary recognition; ACE-III—a developed 100-point test, which allows early differential diagnosis; M-ACE—a shortened 30-point version of ACE-III, feasibly administered within a time frame comparable to MMSE.

#### 2.2.1. Mini-Mental State Examination (MMSE)

MMSE was first published in 1975 [20]. It was standardised and normalised in Poland by a publishing division of the Polish Psychological Association, Pracownia Testów Psychologicznych [21].

#### 2.2.2. ACE-III

ACE-III is an international test used for both overall assessment and selective assessment within five cognitive domains. It is considered a targeted tool meant as a screening assessment of particular dysfunctions or, in other words, used for screening differential diagnoses [3]. The current Polish version was developed in 2015 by Senderecka et al.; Thomas Bak consulted on language and professional issues, and Prof. John Hodges holds the copyrights to the test [22].

ACE-III is a test that assesses the following five cognitive domains: attention, memory, verbal fluency, language and visuospatial abilities, with scores within the following ranges:

- Attention: score 0–18,
- Memory: score 0–26,
- Verbal Fluency: score 0–14,
- Language: score 0–26,
- Visuospatial Abilities: score 0–16.

The test’s structure allows an assessment of particular cognitive domains, as it addresses specific difficulties and deficits.

#### 2.2.3. M-ACE

M-ACE is a shorter version of ACE-III. It is often referred to as mini-ACE or MACE. The test was developed at Neuroscience Research Australia. It is available in various language versions and multiple adaptations, including a Polish version [8,9,22].

### 2.3. Procedure

The assessment procedure was conducted identically for each participant:

1. A partly structured interview was conducted, comprising the educational and professional background, selected aspects of social functioning, health and conditions, which could impact cognitive functions, such as hypothyroidism, cardiovascular diseases, mental disorders and others. Participants with the following conditions were excluded from the study: sense organ disorders (unless participants had the recommended prescriptive glasses or a hearing aid), intractable non-nociceptive pain, suspected but untreated cases of severe depression, psychiatric disorders severe enough to prevent assessment. Participants who displayed any of the symptoms above were referred to a specialist for diagnosis and treatment. People over 60 years of age, capable of linguistic, visual and auditory communication in a stable somatic state, were included in the study. Persons declaring severe somatic complaints or pain, as well as those whose mental state required medical intervention in the first place, were excluded.
2. MMSE and ACE-III screening tests were conducted, and the results were used to calculate the M-ACE results.
3. In accordance with the standards of psychological assessment, each participant was presented with the results of the assessment and further recommendations.

### 2.4. Data Analysis

The results were given as a standard deviation, mean and median; the results were checked for normal distribution. If the results failed to meet normal distribution, the variables were analysed by using nonparametric tests.

The groups were analysed for such characteristics as age, sex and years of schooling. Next, the raw data from each screening test were analysed.

#### 2.4.1. Data Analysis for a Single Assessment to Establish Group Characteristics and Test Results Analysis

Step one of the analysis included 386 people. Personal characteristics were given based on the means, the median and allowing for standard deviation for each variable: age or years of schooling. Next, an effort was made to establish the following relations in the groups under study:

- raw MMSE results and age, sex or years of schooling;
- ACE-III results and age, sex or years of schooling;
- results of M-ACE and age, sex or years of schooling.

#### 2.4.2. Comparing the Sensitivity of the Screening Tests

Step two of the analysis comprised the Spearman’s rank correlation coefficient to compare the sensitivity of each screening test with regard to the assessment of cognitive function in differentiating norms and results below the norm. The following criteria were adopted:

- *r* = 0—no correlation,
- 0 < *r* < 0.2—remote correlation,
- 0.2 ≤ *r* < 0.4—weak correlation,
- 0.4 ≤ *r* < 0.6—average (medium) correlation,
- 0.6 ≤ *r* < 0.8—high (strong) correlation,
- 0.8 ≤ *r* < 1—very high (very strong) correlation,
- *r* = 1—perfect correlation (complete).

The analytics were conducted for:

1. MMSE and ACE-III,
2. MMSE and M-ACE,
3. ACE-III and M-ACE.

Further calculations performed using McNemar’s test were meant to determine the rate of patient migrations between the ranges of the norm and the below the norm results (i.e., below the higher cut-off point, which improves the sensitivity of the test) for each test in comparison with MMSE. Data that showed significant changes in patient migration between the two categories allowed us to differentiate between patients with disorders and healthy participants, assuming a possibly low number of erroneous classifications. Thus, the true positive, true negative, false negative and false positive values were established. Further analysis made use of the ROC curve with the aim to assess the test’s ability to differentiate between the objects with and without a particular feature. For each test, the ROC characteristics were established based on the result category obtained, so as to set the cut-off points of highest sensitivity and specificity. For the purpose of this study, the optimum cut-off point on the ROC curve was developed based on the cost of wrong decisions. The cost of wrong decisions was computed under the assumption that all errors are equally costly.

#### 2.4.3. Analysing Cross-Category Migration—Norms, MCI and Dementia

During the next stage, the analysis aimed to draw comparisons between the tests for the three categories: norm, MCI and dementia. The comparison was conducted to verify whether there occurred a statistically significant discrepancy in cross-category patient migration within the three categories for either scale. Prior calculations were grouped into categories, which differentiated between normal, slightly lower results (which could point to mild cognitive disorders constituting a risk factor for developing dementia) and dementia. Bowker’s test of internal symmetry (also called the Bowker–McNemar test) was used here to verify the hypothesis determining the symmetry of two results of measurements executed twice and a feature, which might have more than two categories (PQStat, ND).

#### 2.4.4. Analysing Lower Cut-Off Domains for ACE-III against the MMSE Norm Range

To establish those areas of cognitive functioning whose lower performance occurs first and, thus, could be an early predictor of the disease, the results of the targeted ACE-III test were compared with the MMSE results for the norm range. As ACE-III consists of several domains, a low score in any of the domains lowers the overall result. The analysis of ACE-III revealed which domains might influence the final outcome more, with patients scoring below the norm threshold in ACE-III, despite being classified as within the norm range by MMSE.

The results were computed using the Mann–Whitney U-test, used to verify a hypothesis determining the insignificance of differences between the medians of the analysed variables, assuming the two distributions of the variables to be quite alike (PQStat, ND). This helped ascertain the data for the particular cognitive domain sub-tests, which scored lower than the norm threshold in ACE-III in patients whose results were within the norm on the MMSE scale.

Step one of the analysis pertained to the discrepancies among the mean scores for the domains of Attention, Speech Fluency, Memory, Language and Visuospatial Ability in people who scored within norm results obtained in MMSE and within norm and below norm results in ACE-III. However, as each domain in ACE-III is characterised by a different scale range, the percentage values were used next. Thus, each scale was assigned a range of 0–100% according to the formula

value in % = score/range × 100% (1)

which yielded percentage differences for the mean scores for each ACE-III domain in relation to scales ranging from 1 to 100%.

## 3. Results

### 3.1. Test Group Profile

In total, 386 people took part in the study, including 302 women (78.2%) and 84 men (21.8%). The mean age was 76.9 ± 8.3 years (77.0; 60.0–97.0). Women were younger than men to a statistically significant degree (W: 76.2 ± 8.3 years (76.0; 60.0–97.0 years) vs. M: 79.5 ± 7.8 years (80.0; 62.0–95 years); *p* < 0.001).

On average, participants declared 13.4 ± 3.2 years of schooling (12.0; 57.2–25.0 years of schooling). The mean length of schooling was comparable for either sex (W: 13.3 ± 3.1 years (12.0; 2.0–20.0 years) vs. M: 13.5 ± 3.4 years (13.0; 6.0–25 years)).

### 3.2. Results for Each Scale

#### 3.2.1. MMSE

MMSE was conducted for all 386 study participants. On average, the overall result was 25.7 ± 4.3 (27.0; 10.0–30.0), with 217 people, that is, 56.2% of participants, scoring in accordance with their age norm. Seventy-nine people, that is, 20.5% of participants, scored below the cut-off point of 26 pts but above 23 pts, a range characteristic of high risk of dementia and—in the case of MMSE—a range assumingly corresponding to mild cognitive disorders. The number of people who scored below 24 pts, that is, below the cut-off point, was 90, constituting 23.3% of all participants. This group is very likely to have dementia.

##### Age and Raw MMSE Data

There was a statistically significant correlation between the age of participants and their score results for MMSE (*r* = −0.494; *p* = 0.0000). The correlation was negative—the older the respondent, the lower the result in MMSE.

Additionally, there occurred a statistically significant difference in MMSE results in patients of varying ages (*p* < 0.0001). The mean scores for MMSE depending on the age group profile are presented in Table 1 below.

As compared to the younger group, the other age groups differed in the mean score for MMSE to a statistically significant degree.

Participants over 80 years of age scored significantly lower than any other age group.

People aged 70 to 75 scored significantly higher than people aged 76 to 80.

**Figure 1:**
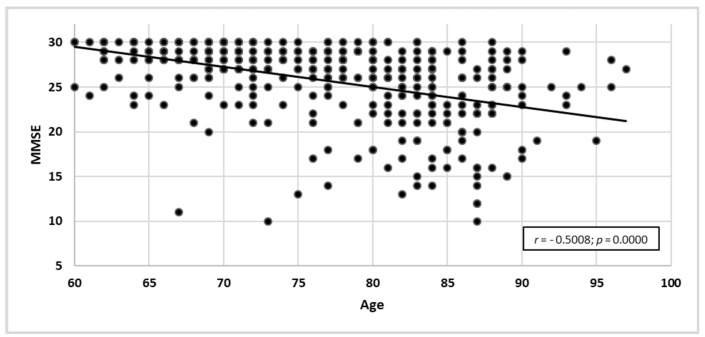
Age correlated with raw MMSE results.

The study showed a negative correlation between the score in MMSE and the age of participants (*r* = –0.5008; *p* = 0.0000). The older the participant, the lower they scored in MMSE (Figure 1).

##### Years of Schooling and MMSE Results

**Figure 2:**
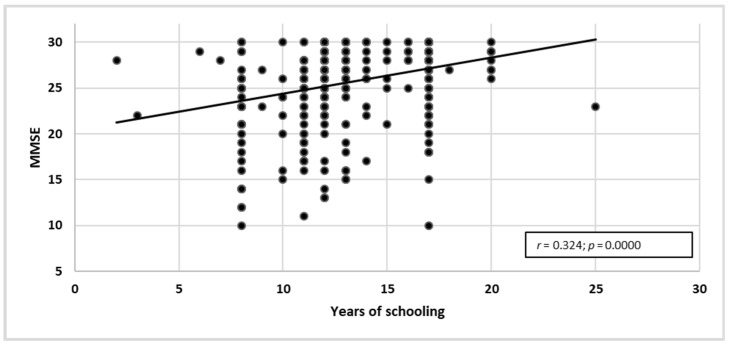
Years of schooling correlated with raw MMSE data.

The study revealed a statistically significant correlation between years of schooling and the MMSE score (*r* = 0.324; *p* = 0.0000). The correlation was positive—the more years of schooling, the better the score in MMSE (Figure 2).

#### 3.2.2. ACE-III

On average, the full-scale ACE-III score (within the range of 0–100 points) was 78.5 ± 16.8 (84.0; 25.0–99.0).

For each component corresponding to a particular domain, the average score was:

- Attention (pts)—15.5 ± 2.9 (17.0; 5.0–18.0), range 0–18 pts;
- Fluency (pts)—9.0 ± 3.5 (9.0; 0.0–25.0), range 0–14 pts;
- Memory (pts)—18.1 ± 6.9 (21.0; 25.0–26.0), range 0–26 pts;
- Language (pts)—22.7 ± 4.0 (24.0; 2.0–26.0), range 0–26 pts;
- Visuospatial Abilities (pts)—13.2 ± 2.6 (14.0; 5.0–16.0), range 0–16 pts.

Among the group tested by means of ACE-III, assuming cut-off points of 88 and 82 points, 150 people, which is 38.9% of the participants, scored within the norm, while 62 people, that is, 16.1%, obtained between 88 and 82 points, which effectively puts them in the range associated with a higher risk of dementia. In clinical terms, this range is characterised by selective or general deficits in cognitive functioning typical for MCI. Lastly, 174 people, which comprises 45.1% of the test group, obtained scores indicating a high likelihood of dementia.

**Figure 3:**
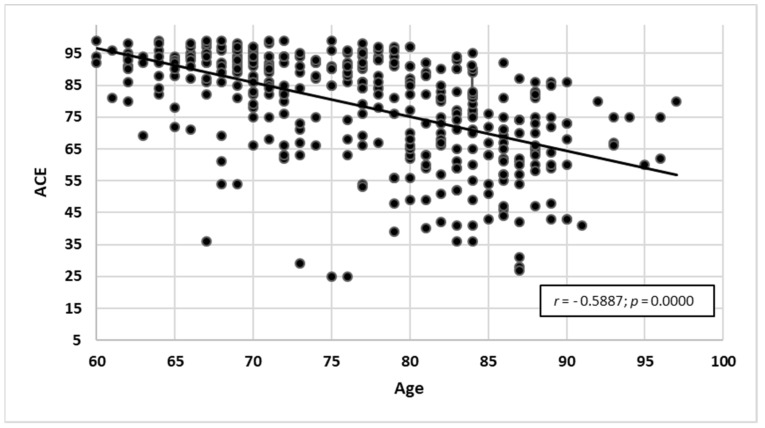
Age correlated with ACE-III test results (*n* = 386).

A negative correlation between ACE-III test results and age was found (*r* = −0,5887; *p* = 0.0000). The older the participants, the lower the ACE-III test results (Figure 3).

**Figure 4:**
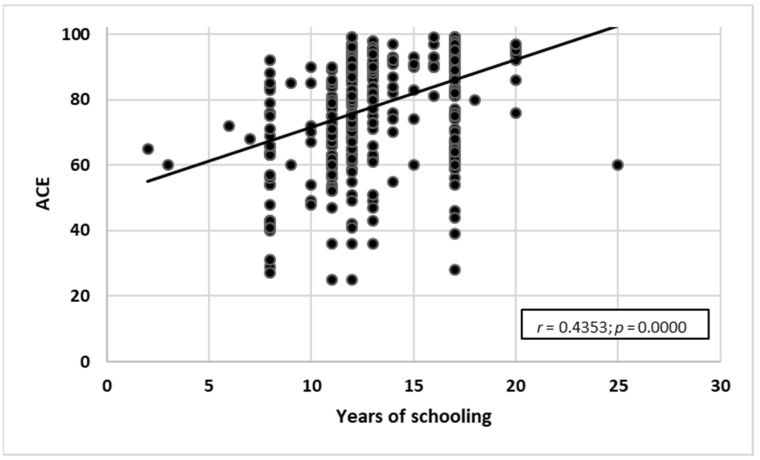
Years of schooling correlated with ACE-III test results (*n* = 386).

The study also yielded a positive correlation between ACE-III test results and years of schooling (*r* = 0.4353; *p* = 0.0000). The higher the number of years of schooling, the higher the score obtained in ACE-III (Figure 4).

#### 3.2.3. M-ACE

On average, the M-ACE test result was 20.8 ± 7.3 (23.0; 1.0–30.0).

Based on the M-ACE test results, assuming cut-off points of 25 and 21 points, as suggested by the test authors considering the validation carried out for the toolkit, 138 people (35.8% of the participants) scored within the norm; 92 people, which amounts to 23.8%, obtained results, which could point to a higher risk of dementia; and 156 people, which is 40.4% of the test group, scored within a range characteristic of a high likelihood of dementia.

**Figure 5:**
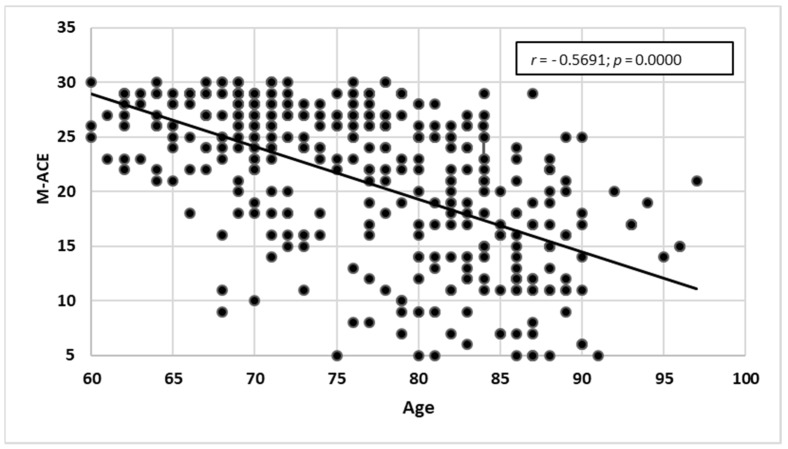
Age correlated with M-ACE test results.

**Figure 6:**
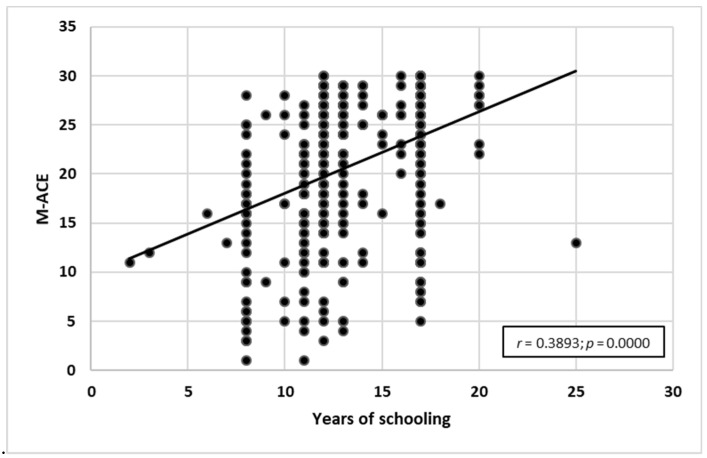
Years of schooling correlated with M-ACE test results.

The research showed a statistically significant negative correlation between age and M-ACE test results (*r* = −0.56914; *p* = 0.0000) and a positive correlation between years of schooling and M-ACE test results (*r* = 0.3893; *p* = 0.0000). The older the participant, the lower they scored in M-ACE. However, the higher the number of years of schooling, the higher the score obtained in M-ACE. Detailed characteristics can be seen in Figure 5 and Figure 6.

#### 3.2.4. Comparing the Scales against MMSE

**Figure 7:**
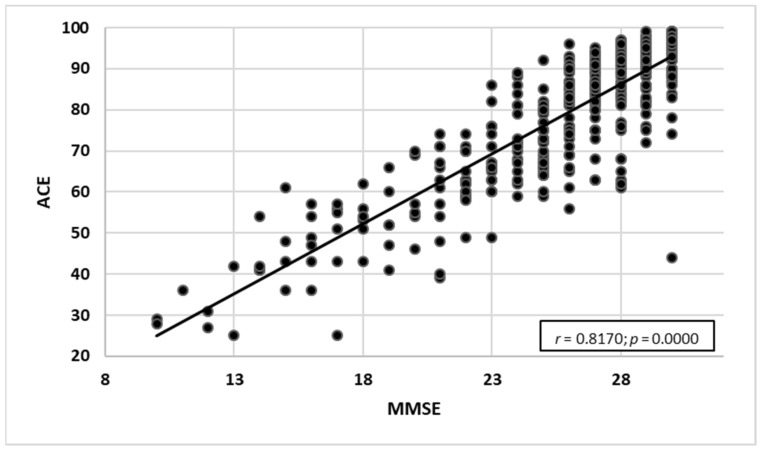
MMSE test results correlated with ACE-III test results (*n* = 386).

**Figure 8:**
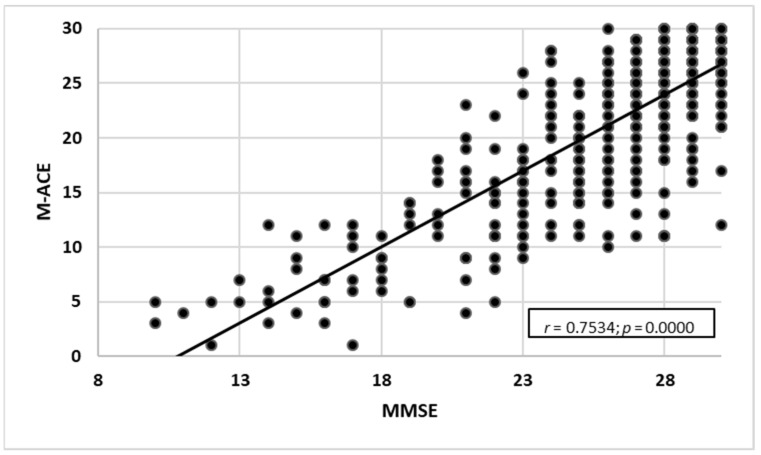
MMSE test results correlated with M-ACE test results (*n* = 386).

**Figure 9:**
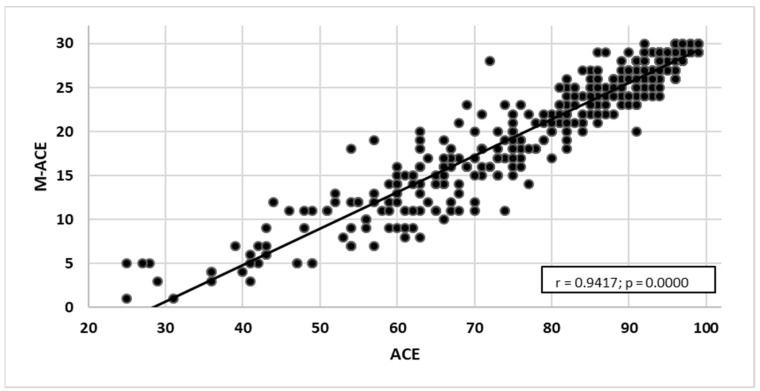
ACE-III test results correlated with M-ACE test results.

An analysis of the rank correlation coefficient for both scales (ACE-III and M-ACE) was carried out to compare the sensitivity of the tests to MMSE raw data. Statistically significant positive correlations were found for the scales:

1. MMSE and ACE-III—a very high correlation with the strength of *r* = 0.817. As the ACE-III test results grew, the MMSE test results increased as well (Figure 7).
2. MMSE and M-ACE—a very high correlation with the strength of *r* = 0.753. As the M-ACE test results grew, the MMSE scores increased as well (Figure 8).
3. ACE-III and M-ACE—a very high correlation with the strength of *r* = 0.942. As the ACE-III test results grew, the M-ACE test results also increased (Figure 9).

#### 3.2.5. ACE-III and MMSE

##### ACE-III and MMSE—Two Categories (below the Norm and Norm)

Among 217 (56.2%) participants who scored within the norm in MMSE (27–30 pts), every third participant (*n* = 77; 35.5%) scored below the norm in ACE-III (0–88 pts). Among 150 (38.9%) respondents whose scores ranged within the norm in ACE-III (89–100 pts), only 10 people (6.7%) scored below the norm in MMSE (0–26 pts).

There were, in total, 299 (159 + 140) people whose test results placed them in the same category (below the norm or norm) on both scales. They accounted for 77.5% (41.2 + 36.3%) of all participants. Ten participants (2.6%) whose MMSE test results placed them within the below-norm category scored within the norm in ACE-III. Meanwhile, 77 people (19.9%) whose MMSE scores categorised them within the norm obtained below-norm scores in ACE-III.

McNemar’s test revealed a statistically significant change in patient cross-category migration between MMSE and ACE-III (χ^2^ = 50.1; *p* < 0.0001). As compared to MMSE, more people (77) crossed over to a lower category than to a higher category (10 people).

The following results were obtained:

- true positive—159,
- false positive—77,
- false negative—10,
- true negative—140.

##### ACE-III—ROC Curve

The area under the ROC curve for the ACE-III diagnostic variable had a very high value (AUC = 0.920 ± 0.014).

**Figure 10:**
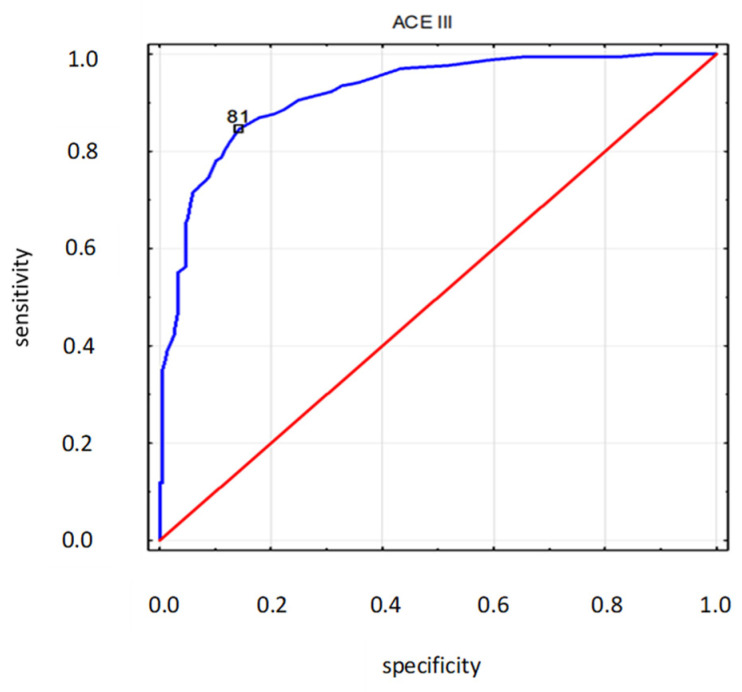
ROC curve (blue) of ACE-III for the MMSE state variable. Diagnostic variable—ACE-III (destimulant); State variable—MMSE: 0—norm (27–30 pts), 1—below the norm (0–26 pts); red—random classifier.

For ACE-III, the suggested cut-off point was 81. For this cut-off point, the costs of erroneous decisions were at a minimum (error rate = 14.8%; for the cut-off point of 88, the error rate was 22.5%). At the cut-off point of 81, the values of sensitivity and specificity reached their peak value (sensitivity = 0.846; specificity = 0.857) (Figure 10).

##### ACE-III and MMSE—Three Categories (Dementia, MCI, Norm)

Considering how significant, in clinical terms, the difference between the category of MCI and the category of dementia is, particularly since it concerns prognosis and the recommended course of treatment, an additional analysis including MCI as a separate category was conducted. Among the study participants, only 62.7% (*n* = 242) obtained scores that placed them within the same category (dementia, MCI or norm) in both ACE-III and MMSE. For 12 respondents (3.1%), the ACE-III category was increased as compared with MMSE.

- Two people (0.5%) whose MMSE results allocated them to the category of dementia received the status of MCI in ACE-III.
- Ten people (2.6%) whose scores assigned them to the category of MCI in MMSE were classified within the norm in ACE-III.
- For 132 respondents (34.1%), the category obtained in ACE-III was lower as compared with MMSE.
- Fifty-five people (14.2%) who were categorised as MCI in MMSE obtained results classified as dementia in ACE-III.
- Thirty-one people (8.0%) whose scores classified them as within the norm in MMSE obtained results classified as dementia in ACE-III.
- Forty-six people (11.9%) whose scores allocated them within the norm in MMSE obtained the status of MCI in ACE-III.

There occurred a statistically significant difference in patient cross-category migration between MMSE and ACE-III classifications (χ^2^ = 98.3; *p* < 0.0001); more people crossed over to a lower category (132 people) than to a higher category (12 people) in ACE-III as compared with MMSE.

##### Analysing Undercut Areas for ACE-III against the MMSE Norm Range

The next stage of the analysis comprised a search for more subtle functioning differences in people whose test results were within the norm in either test and people who scored within the norm in MMSE and below the norm in ACE-III. Thus, people with the measurement executed once and whose MMSE test results ranged between 27 and 30 points were also included in this part of the study (W: *n* = 178; M: *n* = 39). Within this group, there were statistically significant differences observed between people who scored below the norm (0–88 pts) and those within the norm (89–100 pts) for either of the five domains of ACE-III. People whose test results fell within the norm in ACE-II scored higher in the domains of Attention, Verbal Fluency, Memory, Language and Visuospatial Abilities. Table 2 shows more detailed information.

**Figure 11:**
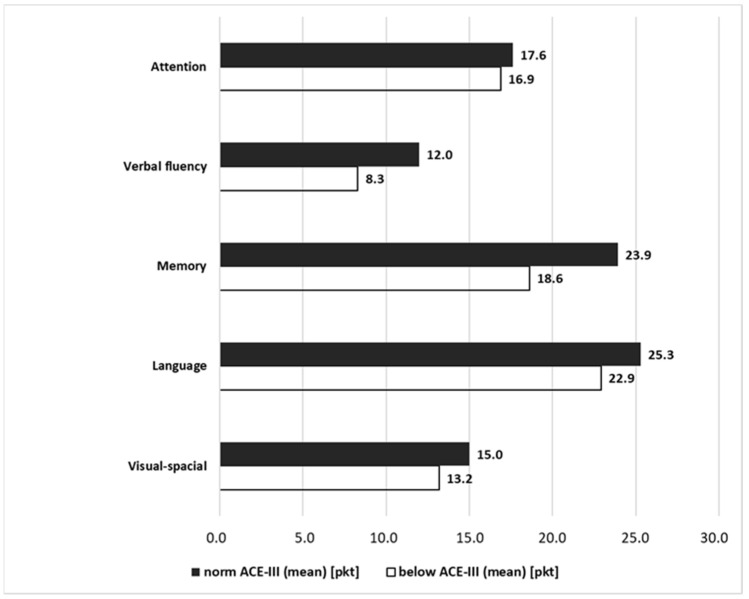
Mean scores as obtained for the five sub-domains by people who scored within the norm and below the norm in ACE-III.

The highest difference in the scores obtained could be observed for the domains of Fluency and Memory, with 3.7 points and 5.3 points, respectively (the mean percentage differences amounted to 26.4 and 20.4%, respectively). Indirectly, the difference between the people within the norm and below the norm could be derived from the Z value of the Mann–Whitney U-test results (Fluency Z = −9.58, whereas Memory Z = −9.53, where the numerical value corresponds to the difference, and the sign “−” corresponds to the direction of change). A detailed description is shown in Figure 11.

Thus, allowing for the differences in mean scores, the domain of Memory presented the highest difference. However, as each domain in ACE-III is characterised by a different scale range, the percentage values were used next. Thus, each scale was assigned a range of 0–100% according to Formula (1) in Section 2.4.4.

This yielded the following results:

- Attention 1 pt = 5.6%,
- Fluency 1 pt = 7.1%,
- Memory/Language 1 pt = 3.8%,
- Visuospatial Abilities 1 pt = 6.3%.

For Fluency, the difference in the mean score was 0.7 points, corresponding to the difference in percentage means of 3.9%. Analogically, for the other domains, the differences in the percentage mean were as follows: Fluency—26.4%; Memory—20.4%; Language—9.2%; and Visuospatial Abilities—11.3%.

**Figure 12:**
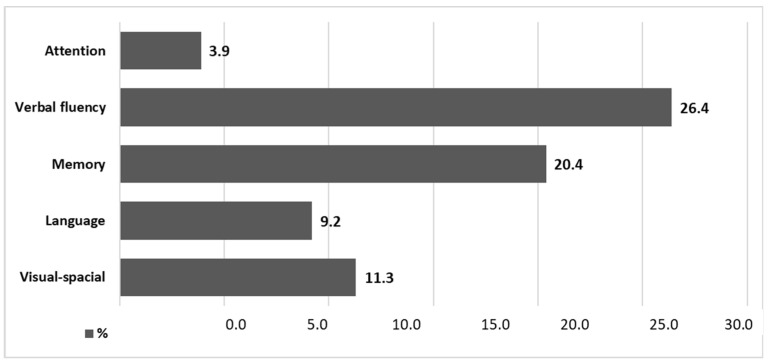
Percentage differences in means between the results within the norm and below the norm for each domain in ACE III (with regard to the scale range of 0–100%).

The domains of Fluency and Memory had the greatest influence on cross-categorising MMSE within the norm scores to scores below the norm in ACE-III. A detailed profile can be found in Figure 12.

#### 3.2.6. M-ACE and MMSE

##### M-ACE (Cut-Off Point of 25) and MMSE—Two Categories (below Norm and within Norm)

Out of 217 (56.2%) participants who scored within the norm in MMSE (27–30 pts), 90 respondents (41.5%) obtained a score below the norm in M-ACE (0–25 pts). Among 138 (35.8%) respondents who scored within the norm in M-ACE (26–30 pts), only 11 people (8.0%) were classified below the norm in MMSE (0–26 pts).

A total of 73.8% (*n* = 285) of study participants obtained scores, which placed them within the same category in both M-ACE and MMSE. One hundred and eleven respondents (2.8%) whose scores categorised them as below the norm in MMSE received the category of within the norm in M-ACE. Meanwhile, 90 people (23.3%) whose MMSE test results placed them within the norm obtained below-norm results in M-ACE.

McNemar’s test revealed a statistically significant change in patient cross-category migration between MMSE and M-ACE (χ^2^ = 60.2; *p* < 0.0001); more people were categorised as lower category (90 people) than higher category (113 people) in M-ACE as compared to MMSE.

The following results were obtained:

- true positive—158,
- false positive—90,
- false negative—11,
- true negative—127.

##### M-ACE—ROC Curve

The area under the ROC curve for the M-ACE diagnostic variable had a very high value (AUC = 0.891 ± 0.017).

**Figure 13:**
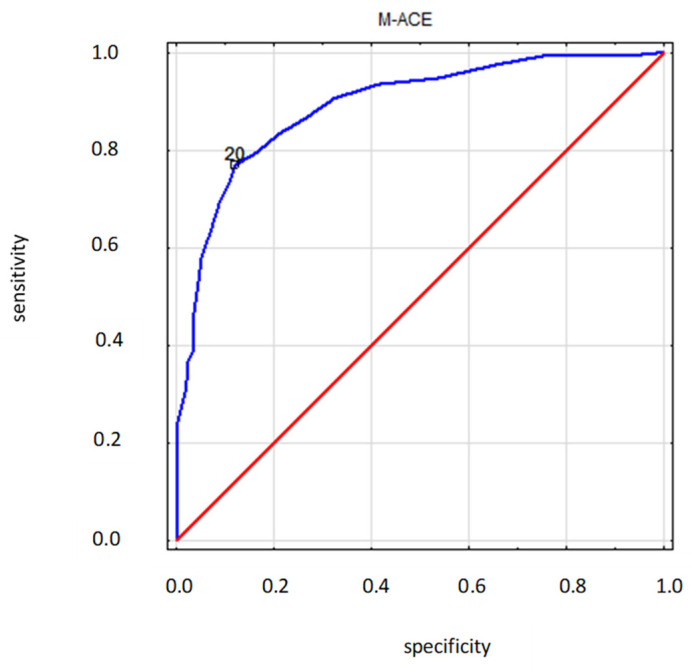
ROC curve (blue) of M-ACE for the MMSE state variable. Diagnostic variable—M-ACE (destimulant); State variable—MMSE: 0—norm (27–30 pts), 1—below norm (0–26 pts). ACE-III and MMSE—three categories (Dementia, MCI, Norm); red—random classifier.

For M-ACE, the suggested cut-off point was 20. For this cut-off point, the costs of erroneous decisions were minimal (error rate = 16.8%; for the cut-off point of 25, the error rate was 26.2%). With a cut-off point of 20, the value for sensitivity was 0.769, while for specificity, it was 0.880; however, neither value reached its peak at this cut-off point. Sensitivity and specificity simultaneously reached their maximum values at the cut-off point of 21 (sensitivity = 0.793; specificity = 0.839; error rate = 18.1%) (Figure 13).

Among the study participants, only 62.7% (*n* = 242) obtained scores that placed them within the same category (dementia, MCI or norm) in both ACE-III and MMSE. For 14 people (3.7%), the category obtained in M-ACE was higher than the category allocated in MMSE:

- Three people (0.8%) who were assigned to the category of dementia in MMSE received the category of MCI in M-ACE;
- One person (0.3%) whom the MMSE test results placed in the category of dementia obtained the status of MCI in M-ACE;
- Ten people (2.6%) whose scores categorised them as MCI in MMSE were allocated within the norm in M-ACE.

Every third person (*n* = 134; 34.7%) had their category lowered in M-ACE as compared to MMSE:

- Forty-four people (11.4%) who were assigned MCI in MMSE obtained the category of dementia in M-ACE;
- Twenty-six people (6.7%) whose scores allocated them to within the norm in MMSE were classified as dementia in M-ACE;
- Sixty-four people (16.6%) who scored within the norm in MMSE received the status of MCI in M-ACE.

The Bowker–McNemar test showed that there occurred a statistically significant change in patient cross-category migration between the MMSE and M-ACE tests (χ^2^ = 93.3; *p* < 0.0001); more people were assigned to a lower category (134 people) than a higher category (14 people) in M-ACE as compared to MMSE.

## 4. Discussion

### 4.1. Notes on the Test Group Profile

Out of 386 people who qualified for the test, 78.2% were women, while men comprised 21.8%. In addition, women were significantly younger than men. Such a distribution reflects the actual percentage of patients in geriatric health centres, especially with regard to psychological diagnostics and the willingness to participate in organised forms of motivation programmes addressed to older people. Apart from demographic factors (such as women’s life expectancy being longer), what also impacts the situation are cultural factors, such as unwillingness to seek professional help in mental disorders, which is connected with a fear of stigma, more often than not observed in older people, and especially men [23]. The other elements thought to uphold the current status quo include factors such as a greater resilience among women, their more open attitude towards showing weakness and seeking help, and a healthier lifestyle as compared to men, as well as being more engaged in upholding social relations, which Livingston et al. [24], in their recent report published in *The Lancet*, considered one of the twelve preventive measures against dementia. Isolation and loneliness are seen as factors conducive to the development of dementia, as, often, these result in impoverished cognitive reserve, limited cognitive stimulation and an increased risk of mood disorders [25]. For instance, a comprehensive 10-year research work in Japan demonstrated that the likelihood of developing dementia in people who scored highest on a five-point social contact scale, including marital relations, family, friends and other social groups, as well as engagement in paid work, was 46% lower than in people with narrow social networks [26].

The study revealed that the test results declined with age; however, it was people under 70 who obtained the highest scores. Additionally, people between 71 and 80 years of age displayed significantly higher test results than people over 80, which supports the claim that problems in engaging the cognitive processes in test-solving tasks increase with age but also result from health conditions in people who apply for help at geriatric health care centres. This line of reasoning finds support in the latest research on the cognitive functioning of the oldest—those over 80 years old [27,28,29].

Education is a crucial factor that impacts cognitive agility in older people. It constitutes an element of the cognitive reserve (CR), which is a cognitive strategy and skill set that allow us to compensate for shortcomings, which stem from neurodegeneration [27,30,31]. This study demonstrated a significant positive correlation: as the number of years of schooling increased, the test results obtained by the MMSE respondents increased. The results reflect our current understanding of the process, where education, especially early education, is listed as one of the main neuroprotective factors [24]. Stephan et al. [28] also presented notable findings. Their study showed that education serves as a neuroprotective factor only in the oldest people who do not display any cognitive disorders or display only mild cognitive disorders; however, it provides no protection in people whose cognitive deterioration process started earlier (before 80). Cognitive disorders tend to advance rapidly in people over 80, regardless of the number of years of schooling [28]. The research, however, requires further verification.

### 4.2. Assessment with the Use of Three Screening Tests

For each assessment with the use of ACE-III and M-ACE, the distribution was calculated based on the score range of the norm, the range below the norm corresponding to MCI and the below-norm range suggestive of dementia. Additionally, the raw data from all three diagnostic tools were correlated, which helped establish high and very high correlations between MMSE and ACE-III (MMSE and M-ACE—high correlation; ACE-III and M-ACE—very high correlation). Consequently, the test results for all three methods are coherent.

Further analysis confirmed that the area under the ROC curve for the ACE-III diagnostic variable had a high value (AUC = 0.920), and a new cut-off point of 81 points was suggested to differentiate between healthy and ill people. The new threshold was lower than the original cut-off point by 1 point, with respect to the bottom cut-off point, and by 7 points, with respect to the top cut-off point. The latest research on Chinese versions of the test suggests a cut-off point of 85, with AUC = 0.978 [32]; meanwhile, recent studies on Portuguese editions suggest a threshold of 82 to differentiate between healthy people and those with MCI, and a threshold of 66 to differentiate between people with MCI and dementia [33]. The emerging differences are explained by means of varied test group profiles and the nature of the institution, which conducted the research. The low cut-off point proposed in this study could be due to the specific character of MMSE, which served as a reference point.

Compared to M-ACE, the area under the ROC curve was also very high (AUC = 0.891), and the suggested cut-off point was 20 points, and it was lower by 1 point, with respect to the original bottom threshold, and by 5 points, with respect to the top cut-off point. Meanwhile, compared to Larner’s suggestions, the cut-off point proposed here was 4 points lower, with respect to the top cut-off point, but equal to the bottom cut-off point [34]. Following Beishon et al. [4], it seems considerably more appropriate to assume lower cut-off points of 82 points for ACE-III and 21 for M-ACE. The results of this study are close to the above-mentioned metanalysis. The occurring differences amount to but one point and could simply be a result of diverse profiles. Still, as Beishon et al. [4] stress, lower thresholds are characterised by higher specificity and acceptable sensitivity, which makes them better suited for clinical use. Similarly, in research utilising the Spanish versions of M-ACE and MMSE, which included low-educated people and mixed dementias, with a control group, the optimum sensitivity (0.867) and specificity (0.870) in M-ACE were observed for the range of 16 to 17 points. The test proved more sensitive than MMSE, simultaneously incurring a lower risk of the floor effect [11]. Here, also, any discrepancies between the results stem from substantial differences in personal profiles. This analysis included people with a broad range of deficits and varied educational backgrounds.

Thus, further analysis was performed based on calculations, which included an additional category: MCI. Research showed that nearly 62% of participants obtained a score within the same category across all three screening tests: ACE-III, M-ACE and MMSE (be it dementia, MCI or norm). In ACE-III, merely 3.1% of participants were moved a category higher, from dementia to MCI or from MCI to within norm, as compared to the MMSE test results. As many as 34.1% of participants had their category lowered in ACE-III as compared to MMSE, with 14.2% crossing from MCI to dementia, 8% having the category shifted from within norm to dementia, and 11.9% being recategorised from within norm to MCI. Similarly, in M-ACE, 34.7% had their category lowered, including as many as 16.6% being shifted from within the norm to MCI. This testifies to the high sensitivity of each test to differentiate between healthy people and those living with a disorder, with ACE-III (to a greater degree) and M-ACE being capable of more detailed ability to differentiate between MCI and healthy population or MCI and dementia. Similar results were reported in analogical research abroad [14,16]. For instance, Senda et al. [17], while verifying the sensitivity of the screening tests, utilised another methodology. The study participants were asked to perform five tests alongside taking part in a range of other diagnostic assessments, including neuroimaging. A body of specialised practitioners examined the cumulative test results in order to establish a diagnosis, only to next verify the sensitivity of the screening tests in question in comparison to the diagnosis given. In light of their research, the sensitivity of ACE-III and M-ACE was far superior to other tests, including MMSE [17]. One advantage of such methodology is the possibility of confronting screening test results and objective diagnosis, which can only be carried out in highly specialised diagnostic centres equipped with appropriate technological and financial resources. What amounts to a disadvantage in this approach was the fact that the assessment by means of all screening tests was performed on the same day, which could have modified the results due to the repetitive character of individual tasks in successive tests. Senda et al. [17] corroborated the differential superiority of ACE-III as compared to MoCA, MMSE or Hasegawa’s test in detecting MCI—however, not so for M-ACE. Research by Larner [13] confirmed the greater sensitivity of M-ACE than MoCA in differentiating MCI, which probably stemmed from some elemental differences in the populations under study, especially as it pertained to average age (in Senda et al. [17], the average age was 73; Larner [13]—59). A study by Miranda et al. [35], on the other hand, paid attention to the memory domain in M-ACE (the component requires the interviewee to repeat and recall three words, a family name and an address), which engages episodic memory. Episodic memory deficits are a highly distinctive symptom of early AD. Tasks on verbal fluency, which activates the executive and linguistic functions, or the clock test, which engages the memory, visuospatial abilities, executive functions and verbal comprehension, and other similar M-ACE components differentiate patients with early deficits typical of dementia in AD and other conditions [35].

Further analysis in this study aimed to identify those ACE-III sub-tests whose results deviated from the norm to a large extent as compared to results within the norm obtained with MMSE. The most dramatic decrease in the mean score values for people who scored below or within the norm of the ACE-III test, as compared to the MMSE norm, occurred for speech fluency (decrease of 26.4%), followed by memory (decrease of 20.4%), visuospatial functioning (decrease of 11.3%), language function (decrease of 9.2%). Attention mean values decreased the least, by only 3.9%. This points to a crucial significance of the task, which forces respondents to search their mental schemata mediated by lexical means under time pressure (which is essentially what the verbal fluency sub-test checks), in identifying the earliest predictors of cognitive deficits. The other telling domain is the mnestic one, which MMSE is hardly equipped to verify. Deficits in either of those cognitive domains, memory or fluency, are essential in dementia onset and development, including the most frequent forms of dementia. These include amnestic AD [36,37], which comprises decreased verbal fluency, specifically, semantic fluency, and reduced episodic memory [38,39] but also other forms, such as frontotemporal dementia, particularly its PPA variants [7,8,40,41]. The literature lacks references to similar studies, which allow for results collected for individual domains using ACE-III—a point raised in a meta-analysis by Beishon et al. [4]. A Polish study presented an analysis of the neuropsychological profiles characteristic of the selected types of dementia [9].

## 5. Conclusions

The results of this study indicate that common knowledge on the characteristics of particular screening tests and their diagnostic applications must be verified. M-ACE seems most advantageous compared to MMSE when it comes to a rapid screening assessment of cognitive functioning. Moreover, adjusted MMSE scoring for education, although useful in clinical practice, as it mitigates the risk of a false positive diagnosis, requires additional information about the patient and professional experience. Targeted ACE-III seems best recommended for use in specialised diagnostic centres as a screening tool [4], whereas M-ACE, which is of higher sensitivity and specificity than MMSE, appears to be a better suited diagnostic alternative for primary health care centres.

## Figures and Tables

**Table 1:** Descriptive characteristics for MMSE depending on the age group and test results for the Kruskal–Wallis test.

Age	*n*	MMSE [pts]	H	*p*	
up to 70 years old [G1]	108	28.0 ± 2.7 (29.0; 11–30)	97.6	0.0000	
71–75 years old [G2]	60	27.0 ± 3.7 (28.0; 10–30)			
76–80 years old [G3]	73	26.2 ± 3.6 (27.0; 14–30)			[G1] *p* = 0.0009
over 80 years old [G4]	145	23.3 ± 4.6 (24.0; 10–30)			[G1] and [G2] *p* < 0.0001 [G3] *p* = 0.0001

**Table 2:** Detailed profile of the five sub-domains of the study participants who scored below and within the norm in ACE-III as well as Mann–Whitney U-Test results (only people who scored within the norm in MMSE).

Scale	ACE-III	*n*	Mean, SD, Median, Range	Mann–Whitney U-Test	
				Z	*p*
Attention (pts)	below	77	16.9 ± 1.4 (17.0; 11–18)	−4.46	0.0000
	norm	140	17.6 ± 0.7 (18.0; 15–18)		
Verbal Fluency (pts)	below	77	8.3 ± 2.3 (9.0; 2–12)	−9.58	0.0000
	norm	140	12.0 ± 2.2 (12.0; 6–25)		
Memory (pts)	below	77	18.6 ± 4.6 (20.0; 7–25)	−9.53	0.0000
	norm	140	23.9 ± 2.3 (24.0; 9–29)		
Language (pts)	below	77	22.9 ± 2.5 (24.0; 15–26)	−8.55	0.0000
	norm	140	25.3 ± 1.0 (26.0; 21–26)		
Visuospatial Abilities (pts)	below	77	13.2 ± 2.2 (13.0; 5–16)	−6.92	0.0000
	norm	140	15.0 ± 1.2 (15.0; 10–16)		

ACE-III—below norm: 0–88 pts; within norm: 89–100 pts.

## Data Availability

Data are available from the corresponding author upon reasonable request.
